# Supplementary material for: Content-rich biological network constructed by mining PubMed abstracts
Source: BMC Bioinformatics. 2004 Oct 8;5:147. doi: 10.1186/1471-2105-5-147 (PMC528731; doi:10.1186/1471-2105-5-147)
Supplement: Additional File 2 — The original results of the above study (non-essential files are deleted to keep the file size under the limit set by BMC bioinformatics). [file 1471-2105-5-147-S2.bz2 › chilibotAdditionalFile2/dip05/615ID9219685E10999/html/B2M_CD1D1.html]

 


 **B2M** and **CD1D1** 
  
Found 16 abstracts in PubMed, retrieved 05.  
 

 What does Google say? 
 PDF only 
| .edu only 

---

**Interactive relationship** (e.g. stimulation, inhibition, etc)

**Neutral relationship**- We demonstrate that chaperone and foldase assisted refolding of denatured CD1d  [ **CD1D1** ]  molecules and beta 2 microglobulin  [ **B2M** ]  in the presence of synthetic lipids is a rapid method for the generation of functional and specific CD1d  [ **CD1D1** ]  tetramers, which unlike previously published protocols ensures isolation of CD1d  [ **CD1D1** ]  tetramers loaded with a single lipid species.  Ref: 11248072 Proc Natl Acad Sci U S A, 2001
- The biochemical structure of CD1d  [ **CD1D1** ]  on intestinal epithelial cells IECs exists in two forms a 37 kD nonglycosylated, beta 2 microglobulin  [ **B2M** ]  beta 2M independent and a 48 kD glycosylated, beta 2M dependent form.  Ref: 11296664 Keio J Med, 2001
